# Optogenetic manipulation of cGMP in cells and animals by the tightly light-regulated guanylyl-cyclase opsin CyclOp

**DOI:** 10.1038/ncomms9046

**Published:** 2015-09-08

**Authors:** Shiqiang Gao, Jatin Nagpal, Martin W. Schneider, Vera Kozjak-Pavlovic, Georg Nagel, Alexander Gottschalk

**Affiliations:** 1 Department of Biology, Institute for Molecular Plant Physiology and Biophysics, Biocenter, Julius-Maximilians-University of Würzburg, Julius-von-Sachs-Platz 2, D-97082 Würzburg, Germany; 2 Buchmann Institute for Molecular Life Sciences, Goethe University, Max von Laue Strasse 15, D-60438 Frankfurt, Germany; 3 Department for Biochemistry, Chemistry and Pharmacy, Institute of Biochemistry, Goethe University, Max von Laue Strasse 9, D-60438 Frankfurt, Germany; 4 Department of Microbiology, Biocenter, Julius-Maximilians-University of Würzburg, Am Hubland, D-97074 Würzburg, Germany; 5 Cluster of Excellence Frankfurt—Macromolecular Complexes (CEF-MC), Goethe University, Max von Laue Strasse 15, D-60438 Frankfurt, Germany

## Abstract

Cyclic GMP (cGMP) signalling regulates multiple biological functions through activation of protein kinase G and cyclic nucleotide-gated (CNG) channels. In sensory neurons, cGMP permits signal modulation, amplification and encoding, before depolarization. Here we implement a guanylyl cyclase rhodopsin from *Blastocladiella emersonii* as a new optogenetic tool (BeCyclOp), enabling rapid light-triggered cGMP increase in heterologous cells (*Xenopus* oocytes, HEK293T cells) and in *Caenorhabditis elegans*. Among five different fungal CyclOps, exhibiting unusual eight transmembrane topologies and cytosolic N-termini, BeCyclOp is the superior optogenetic tool (light/dark activity ratio: 5,000; no cAMP production; turnover (20 °C) ∼17 cGMP s^−1^). Via co-expressed CNG channels (OLF in oocytes, TAX-2/4 in *C. elegans* muscle), BeCyclOp photoactivation induces a rapid conductance increase and depolarization at very low light intensities. In O_2_/CO_2_ sensory neurons of *C. elegans*, BeCyclOp activation evokes behavioural responses consistent with their normal sensory function. BeCyclOp therefore enables precise and rapid optogenetic manipulation of cGMP levels in cells and animals.

## Abstract (web summary)

Cyclic guanosine monophosphate (cGMP) is a cellular second messenger involved in many processes including regulation of neuronal excitability and vascular tone. Gao, Nagpal *et al*., employ a fungal rhodopsin to optogenetically control cGMP levels in multiple systems including *C. elegans* sensory neurons.

## Graphical abstract

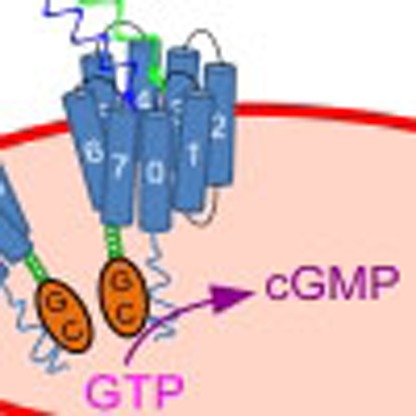

Optogenetic tools developed in recent years opened new avenues in neuro- and cell biological research^1^,^2^,^3^,^4^. These tools allow rapid, reversible and non-invasive modulation of membrane potential, protein–protein interaction, gene expression and second messenger signalling in live cells and whole animals, in a cell-specific manner. Many optogenetic tools are based on microbial rhodopsins^5^. Rhodopsins are membrane proteins, responsible for light perception in the ultraviolet to visible spectral range^6^,^7^. They are composed of a non-absorbing protein, opsin, to which a chromophore, retinal, is covalently linked. Rhodopsins from the animal visual system (type II or metazoan opsins) are G-protein-coupled receptors^8^; they sense light and, in vertebrate rods and cones, via G_o_ signalling-induced hydrolysis of cyclic GMP (cGMP), yield membrane hyperpolarization by closure of cyclic nucleotide-gated (CNG) cation channels^9^. Also some opsins signalling via G_q_ and G_s_ have been described^10^. In *Drosophila* eyes, rhodopsin leads to membrane depolarization via the activation of transient receptor potential (TRP) cation channels^11^.

Several type I (microbial) rhodopsins were discovered and extensively studied, such as the light-activated H^+^ pump bacteriorhodopsin^12^, the Cl^−^ pump halorhodopsin^13^, the sensory rhodopsins^14^, and most recently the channelrhodopsins (ChRs)^15^,^16^. These light-activated channels and pumps were prominently established as optogenetic tools for membrane de- or hyperpolarization, respectively^17^,^18^,^19^. In addition, large microbial rhodopsins were predicted from the *Chlamydomonas reinhardtii* genome sequence, consisting of opsins fused to signalling modules, like nucleotidyl cyclase domains; yet none of these opsins has been experimentally proven to be functional^20^.

Other optogenetic tools utilize LOV or BLUF domains for light sensation and relay photon absorption to different protein domains, thus achieving cellular effects, for example, unmasking protein interaction modules, or triggering enzymatic activity. Optogenetic tools for light-driven cAMP generation exist, for example, photoactivated adenylyl cyclases (PACs), which permit rapid production of cAMP in cells and animals^21^,^22^,^23^, and can affect behaviour through modulation of specific cellular processes or signalling in the nervous system^24^. Another second messenger widely utilized is cGMP, activating protein kinase G, or CNG channels, especially in sensory neurons in various animals. Here guanylyl cyclases act downstream of sensory receptors, or are modularly connected to them, signaling, for example, the detection of light, oxygen, temperature or chemicals. Guanylyl cyclases thus relay primary sensory signals to downstream cellular machinery that encodes this information for a signal output. As the output of a sensory neuron is transmitter release, this can be addressed by optogenetics using ChR2. Yet, intracellular encoding of the primary sensory signal via cGMP, which can be a control point for signal amplification or modulation, cannot be addressed using ChR2. Thus, an optogenetic tool affecting cGMP levels in such cells would enable control over intracellular encoding events in cGMP mediated signalling.

The existing PAC of *Beggiatoa* was converted into a guanylyl cyclase by mutating three amino acids (aa) relevant for nucleotide specificity^23^; the respective protein (named bPGC, BlgC or EROS) has recently been used in male rats, to evoke penile erection, following transfection and blue illumination of the *Corpora cavernosa*^25^. Yet, as bPGC was generated by mutation and did not evolve, its efficiency was low compared with the parental enzyme, and its productivity for GTP over ATP was only ∼7:1 *in vitro*^23^. Thus, naturally evolved light-triggered guanylyl cyclases may be more efficient optogenetic tools.

Recently, a new type I rhodopsin, fused with a guanylyl cyclase domain, was found in the fungus *Blastocladiella emersonii*^26^. This rhodopsin guanylyl cyclase, BeGC1, was shown to be essential for phototaxis of zoospores in *B. emersonii* through green light-regulated cGMP production. Yet, the light-regulated guanylyl cyclase activity of BeGC1 was not directly demonstrated.

Here we show light-regulated guanylyl cyclase activity of BeGC1 by *in vitro* and *in vivo* assays using expression in *Xenopus* oocytes, HEK293T cells and muscle cells of the nematode *Caenorhabditis elegans*. We thus suggest renaming this protein to Cyclase Opsin (guanylyl cyclase opsin from *B. emersonii*; BeCyclOp). Light-activated BeCyclOp potently produces cGMP with high specificity (no cAMP production), fast kinetics and high turnover, and shows highest efficiency among four other fungal CyclOps tested. We co-express BeCyclOp in *C. elegans* muscle cells, together with the *C. elegans* CNG channel comprising TAX-2 and TAX-4 subunits^27^,^28^, to demonstrate light-driven depolarization upon photoactivation of BeCyclOp. Last, in O_2_/CO_2_ sensory neurons of *C. elegans*, BeCyclOp light-activation triggers rapid tactic behavioural responses, which depend on the presence of all-trans retinal (ATR). Thus, BeCyclOp is a potent optogenetic tool for control or modulation of cGMP signalling in cells and animals.

## Results

### Expression of fungal CyclOp proteins in *Xenopus* oocytes

The full-length cDNA and protein product of BeCyclOp has been confirmed in *B. emersonii*^26^. BeCyclOp (626 residues, calculated 68 kDa) comprises a microbial (type I) opsin domain near the N-terminal and a guanylyl cyclase domain in the C-terminal part. Compared with other microbial opsins, BeCyclOp has a much longer N terminus (Supplementary Figs 1 and 2). Transmembrane helix prediction indicates that BeCyclOp might have eight transmembrane helices, while all other opsins known so far are seven transmembrane proteins with extracellular N and cytoplasmic C termini, respectively. The N terminus of BeCyclOp appears to contain no signal sequence, and should thus be cytosolic, like the predicted C terminus. The first transmembrane helix is predicted to span aa 147–169. The fourth helix (aa 249–270) in BeCyclOp shows high conservation with transmembrane helix 3 in other type I opsins (Supplementary Fig. 1, inset). The conserved lysine for binding retinal is in the last, the predicted eighth helix. Avelar *et al*.^26^, identified four additional predicted CyclOps in fungal genomes: three in *Allomyces macrogynus* (AmCyclOp1, 2 and 3) and CaCyclOp in *Catenaria anguillulae* (Supplementary Fig. 2). All of them exhibit the unusual transmembrane domain architecture, including the first transmembrane helix as a specific addition. We thus suggest numbering the CyclOp transmembrane helices from 0 to 7.

**Figure 1:**
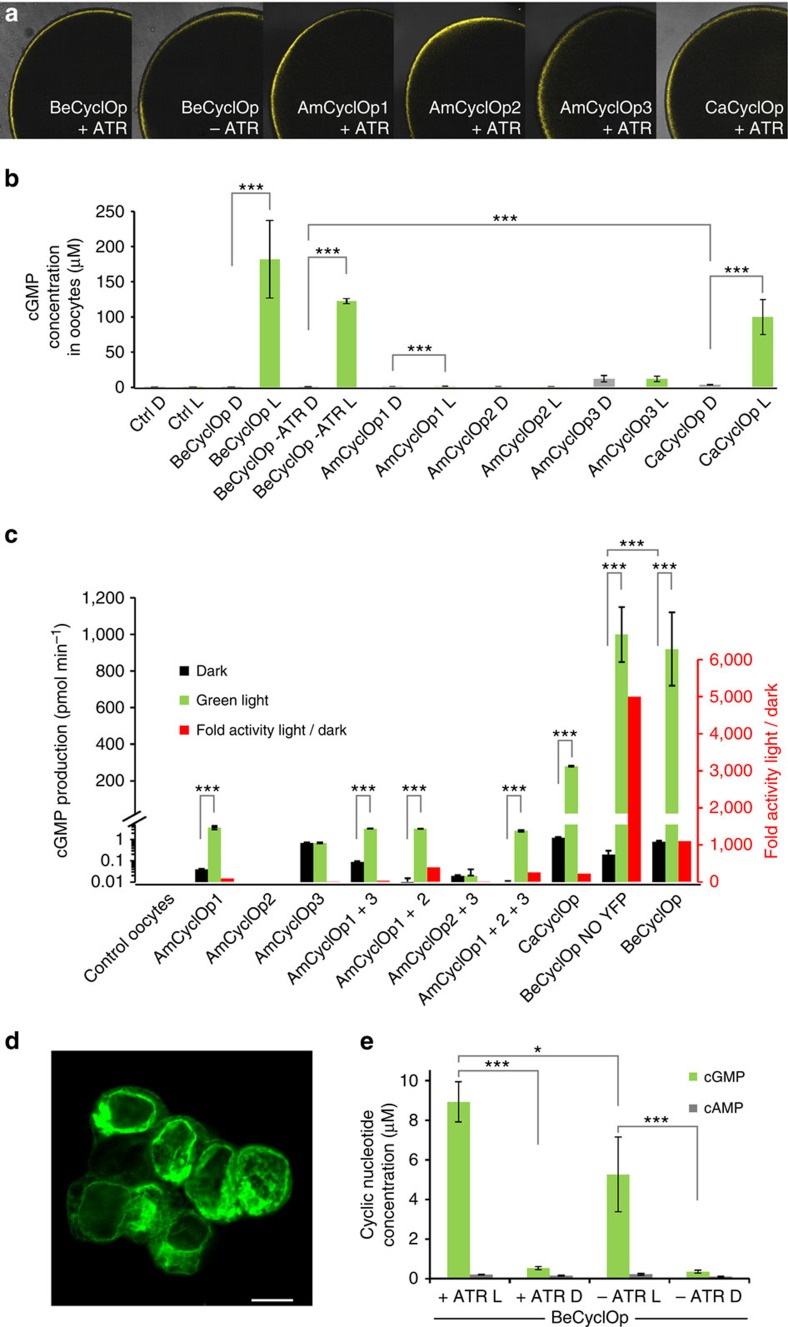
Assessing fungal CyclOps for light-regulated cGMP generation. (**a**) Plasma membrane fluorescence of *Xenopus* oocytes expressing BeCyclOp, AmCyclOp1, 2 and 3, and CaCyclOp, as C-terminal YFP fusions, with and without 1 μM ATR, as indicated. (**b**) cGMP production of different fungal CyclOps (as indicated), fused to YFP, was assessed in extracts prepared from oocytes expressing these proteins, that were either kept in the dark (D), or after 2 min green light (L; 532 nm, 0.15 mW mm^−2^ – BeCyclOp) or 0.5 mW mm^−2^ (all other CyclOps). ATR was added, unless noted. *N*=2 experiments, mean of 5–6 oocytes each, error bars, s.d. (**c**) Light-induced cGMP production in membrane preparations from oocytes expressing various fungal CyclOps as YFP fusions (unless otherwise noted), or combinations thereof (see Tab. 1 for amounts of cRNAs injected). Membranes were kept in dark (D, black bars) or illuminated (L, green bars) with 532 nm light (0.5 mW mm^−^^2^), and parallel reactions were quenched after 1 or 5 min, and cGMP measured. Note the split left *y* axis; logarithmic scale in lower part. On the right *y* axis, and shown in red, is the L/D ratio of the cGMP production rates. Average of *N*=3 experiments; error bars, s.d. Statistically significant differences were determined by one-way analysis of variance: *P<0.05; **P<0.01; ***P<0.001. (**d**) HEK293T cells were seeded on coverslips and transfected with a plasmid encoding BeCyclOp::YFP. Cells were fixed and analysed by confocal microscopy. Scale bar, 10 μm. (**e**) cGMP and cAMP assay of HEK293T cells transfected with BeCyclOp under different conditions.+ATR: 1 μM ATR added;—ATR: no additional ATR. L, in light. D, in dark. Statistically significant differences determined by 1-way ANOVA: *P<0.05; ***P<0.001. *n*=6, error bars, s.d.

We obtained synthetic genes for all five CyclOps, codon optimized to mouse, and expressed them in *Xenopus* oocytes, as C-terminal yellow fluorescent protein (YFP) fusions (CyclOp::YFP). All five CyclOp::YFPs showed robust and similar expression in the dark 3 days post injection (dpi) of in vitro synthesized mRNA (cRNA), as judged by the observable YFP fluorescence in the plasma membrane (Fig. 1a). To assess these cells for light-mediated cGMP generation, the injected oocytes were illuminated for 2 min with green light (532 nm; 0.15 or 0.5 mW mm^−2^), followed by extract preparation and a colorimetric assay for cGMP content (Fig. 1b; Supplementary Table 1). Light-treatment led to a ∼180-fold increased level of cGMP for BeCyclOp::YFP, compared with the extract prepared from control oocytes kept in the dark (*N*=2 experiments, *n*=6 oocytes each). Addition of 1 μM ATR to the oocyte medium led to slightly increased BeCyclOp::YFP expression (Fig. 1a); possibly, binding of ATR to BeCyclOp stabilizes the protein in the oocyte, as previously observed for ChR2 (ref. 29). Light-triggered cGMP production was also slightly higher with ATR (∼1.5-fold; *N*=2 experiments, *n*=6 oocytes each; Fig. 1b; Supplementary Table 1). The *in vivo* assay clearly demonstrated the light-activated guanylyl cyclase activity of BeCyclOp; however, due to variations of the oocytes, and as they contain phosphodiesterases (PDEs) that may degrade the newly generated cGMP, accurate quantification is difficult. We thus designed an *in vitro* assay based on isolated oocyte membranes. Measurements at long times after a light flash showed no decrease in cGMP, thus demonstrating a complete absence of PDE activity in our *in vitro* assay (Supplementary Fig. 3). To compare the different CyclOp::YFPs, we determined their expression levels in oocytes by calibrating the amount of the respective protein via its fluorescence, comparing it with a standard of purified YFP (Supplementary Fig. 4). Expression levels were essentially comparable for distinct batches of oocytes. Therefore, we could reliably determine guanylyl cyclase activities in saturating light (L) and in the dark (D), allowing to determine a L/D ratio of guanylyl cyclase activity (Fig. 1c; Table 1). At 5,000±15-fold (*N*=3), the L/D ratio of BeCyclOp (NO YFP) was highest. Interestingly, L/D appeared fourfold reduced for the fusion protein BeCyclOp::YFP, which showed the same high activity in the light, but a fourfold increased activity in the dark. To verify the enzyme specificity, we assayed BeCyclOp also for cAMP production in these samples; however, no obvious cAMP increase was detected (Table 1), suggesting that BeCyclOp is a highly specific guanylyl cyclase.

CaCyclOp has the highest homology to BeCyclOp, and it also showed by far the highest activity, after BeCyclOp, that is, ∼28–54% of BeCyclOp cGMP production in the light (depending on the assay, *N*=3), together with a slightly increased dark activity (Fig. 1). CaCyclOp thus has a L/D ratio of 230, which is significantly less than the L/D of >1,000 for BeCyclOp or BeCyclOp::YFP. CaCyclOp is the only CyclOp for which we could measure an adenylyl cyclase (AC) activity, however, it was only detectable in light and was <0.1% of guanylyl cyclase activity (*N*=3; Table 1). In contrast, AmCyclOp1 showed very weak, and AmCyclOp2 showed virtually no photoactivated guanylyl cyclase activity, in either assay. AmCyclOp3 led to ∼20-fold higher guanylyl cyclase activity than controls (*N*=3), but this elevated cGMP concentration was also observed in the dark. No obvious AC activity was found under either condition. These proteins belong to class III cyclases, which form homo- or heterodimers^30^,^31^. Therefore, we performed co-injections with different combinations of cRNA for these three CyclOps. AmCyclOp1 and 2 were mixed 1:1. For AmCyclOp3, only 1/10th was used to increase the probability of (the non-light-responsive) AmCyclOp3 to form a heterodimer with AmCyclOp1 or 2. Nearly all different combinations showed some light-gated guanylyl cyclase activity in oocytes, however, at much lower levels than BeCyclOp (generally <1%). The more accurate *in vitro* assay indicated that AmCyclOp1 and AmCyclOp2 could possibly form a heterodimer and function with higher (light) and lower (dark) activity than the AmCyclOp1 homodimer (L/D=400), and AmCyclOp1 could possibly dimerize with AmCyclOp3 (*N*=3; Fig. 1c; Table 1). Further analysis, involving the fungus, is required to show how these three CyclOps function *in vivo*. Among the five CyclOps tested, BeCyclOp qualifies best to be used as an optogenetic tool with high light-activation and weak dark activity. We assessed its functionality in two additional expression systems. In HEK293T cells, transiently expressing BeCyclOp after transfection (Fig. 1d), blue light induced ∼15- to 16-fold cGMP production compared with the dark (with or without addition of ATR; Fig. 1e), while for control cells (light and dark condition), all samples had a cGMP concentration of ∼0.02 μM (+ATR light: 0.024 μM; +ATR dark: 0.02 μM; −ATR light: 0.016 μM; −ATR dark 0.02 μM, all *N*=6). No obvious cAMP production was found. In *C. elegans* extracts, from animals expressing BeCyclOp in muscle cells, likewise a ∼12-fold increase in cGMP production was observed, with no significant cAMP increase (*N*=6; Fig. 4c). We thus characterized the BeCyclOp protein further.

### Turnover of BeCyclOp

We measured the turnover number of BeCyclOp by determining the amount of BeCyclOp::YFP in membranes, based on the YFP fluorescence, and relating it to the amount of cGMP generated in the light (Supplementary Fig. 4). For this experiment, on average, we obtained about 1 pmol of BeCyclOp::YFP per oocyte. *In vitro* assays with these membranes, from 2 different batches of 20 and 21 oocytes (*n*=6), yielded an average 17±4 pmol cGMP per second and per oocyte-derived membrane (equivalent to 1 pmol BeCyclOp; Supplementary Fig. 4). The turnover of BeCyclOp (in light, at 20 °C) is thus ∼17 cGMP s^−1^.

### Action spectrum of BeCyclOp

**Figure 2:**
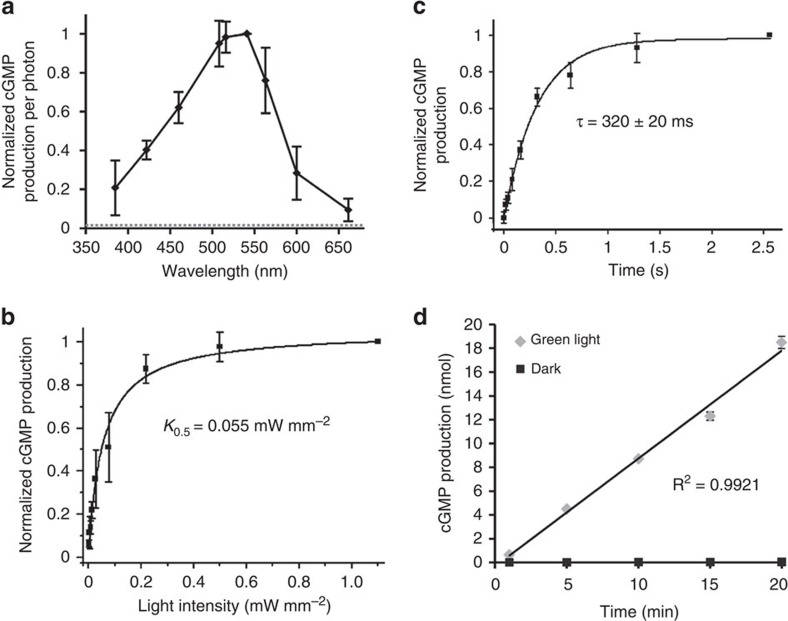
BeCyclOp photoactivation and cGMP production. (**a**) BeCyclOp action spectrum. Dotted grey line indicates the relative dark activity, light intensities at different wavelengths were adjusted to ∼0.02 mW mm^−2^. cGMP production per photon amount was calculated, normalized to the action spectrum peak. Mean of *N*=3 experiments, error bars, s.d. (**b**) Light (532 nm) intensity dependence of mean normalized cGMP production of BeCyclOp-containing membranes; K_0.5_=0.055 mW mm^−2^. *N*=3 experiments, error bars, s.d. (**c**) Kinetics of mean, normalized cGMP production of BeCyclOp-containing membranes, measured at indicated times after 20 ms illumination. Fitting a mono-exponential yields τ=320±20 ms. *n*=5 experiments; error bars, s.d. (**d**) Mean BeCyclOp cGMP production in the dark and with continuous illumination (0.5 mW mm^−2^, 532 nm), measured at indicated times. *N*=3 experiments, error bars, s.d.

To obtain an action spectrum, aliquots of the BeCyclOp-containing membrane preparation were illuminated with light of different wavelengths, but equal amount of photons, and cGMP production was quantified (*N*=3; Fig. 2a). The action spectral peak was around 530 nm (green light), while 660 nm (red) and 385 nm (ultraviolet) light could still activate BeCyclOp, albeit much less efficiently. This is in good agreement with the effects of different light wavelengths in promoting phototaxis of *B. emersonii* zoospores^26^.

### Light intensity dependence of BeCyclOp function

Next, we measured light intensity dependence of BeCyclOp using a green laser (532 nm), near the action spectrum peak. Half-maximal activation (*K*_0.5_) was observed at 0.055 mW mm^−2^ (*N*=3; Fig. 2b). For some previously analysed PACs, cyclase activity continues in the dark after a light flash because of a slow photocycle. For bPAC from *Beggiatoa*, cAMP production levels off with a time constant *τ*=23±2 s at pH 7.4 after a 4 s light flash^22^. The slow photocycle of bPAC is in accordance with a high light sensitivity (*K*_0.5_=4 μW mm^−2^). Similarly, *Microcoleus* mPAC (*K*_0.5_=6 μW mm^−2^) has a slow photocycle (*τ*∼14 s; ref. 32). The larger *K*_0.5_ value for BeCyclOp suggests a faster photocycle.

### Time dependence of light-activated BeCyclOp activity

To measure the life time of the light-induced intermediate of BeCyclOp that possesses the increased guanylyl cyclase activity, we built a computer-controlled system where cyclase activity was initiated by a 20 ms laser flash (532 nm), and stopped after different time intervals (20 ms to 2.6 s) by quenching with 0.1 M HCl. Figure 2c shows that the cGMP concentration keeps increasing in the dark after the light flash with *τ*=320±20 ms (*N*=3) at 21 °C, reaching saturation after ∼1.5 s. Further measurements were extended up to 10 min (Supplementary Fig. 3); however, no ongoing increase or decrease of cGMP concentration in the dark was observed in our *in vitro* assay, thus indicating that indeed the active photointermediate of BeCyclOp is rather short lived, which makes this a well-controllable tool for optogenetics. In continuous light, BeCyclOp catalysed cGMP at a constant rate, showing no deactivation even after 20 min (Fig. 2d).

### The long cytosolic N terminus of BeCyclOp

**Figure 3:**
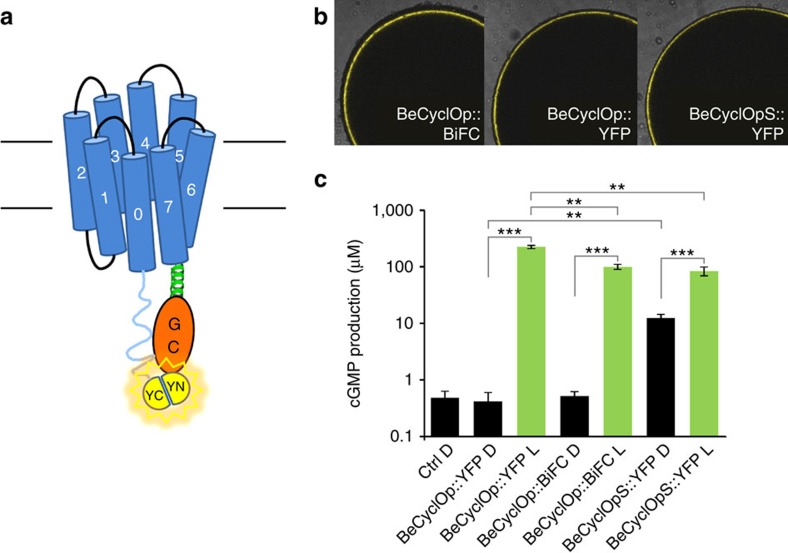
Cytosolic localization and regulation of cyclase activity by the BeCyclOp N terminus. (**a**) BeCyclOp BiFC construct: aa 1–155 of YFP (YN) fused to C-terminal, and aa 155–239 of YFP (YC) fused to the N-terminal ends of BeCyclOp, respectively. (**b**) Oocyte membrane fluorescence images, resulting from expression of different BeCyclOp YFP/BiFC fusion constructs. BeCyclOpS refers to deletion of aa 1–90 in the N terminus. (**c**) cGMP production in *Xenopus* oocyte expressing different BeCyclOp constructs in dark (D) and after illumination (L; 532 nm, 0.2 mW mm^−2^, 2 min). *N*=2 experiments, mean value of six oocytes each; error bars, s.d. Statistically significant differences determined by one-way analysis of variance: **P<0.01; ***P<0.001.

Based on the primary sequence and derived structural model (Supplementary Fig. 1), BeCyclOp (like the other CyclOps; Supplementary Fig. 2) has a cytoplasmic N terminus of 146 aa and a non-canonical transmembrane 0 helix, followed by the canonical seven transmembrane helices of microbial rhodopsins. This is a striking difference to all other type I opsins studied to date. We generated a terminally truncated version of BeCyclOp by cutting the first 90 aa, using the second methionine as translational start. The truncated version (BeCyclOpS) expressed well in *Xenopus* oocytes (Fig. 3b) and exhibited potent light-activated guanylyl cyclase activity (Fig. 3c). However, compared with full-length BeCyclOp, BeCyclOpS shows higher dark activity and lower light-induced cGMP production, suggesting that the BeCyclOp N terminus is required for tight light regulation of guanylyl cyclase activity, further indicating its cytoplasmic localization. To probe the cytoplasmic localization of the N terminus, we used Bimolecular Fluorescence Complementation (BiFC)^33^. To this end, we fused the C-terminal 85 aa (positions 155–239) of YFP (YC) to the N terminus of BeCyclOp and the N-terminal 155 aa of YFP (YN) to its C terminus (BeCyclOp::BiFC; Fig. 3a). Strong fluorescence was observed (Fig. 3b), indicating YC and YN reconstitution of YFP, thus proving cytoplasmic localization of both BeCyclOp termini. Unlike for BeCyclOpS, guanylyl cyclase activity in the dark was not increased in BeCyclOp::BiFC and was only slightly weaker than for BeCyclOp::YFP during illumination (Fig. 3c).

### Activation of a CNG channel after BeCyclOp photostimulation

We previously measured the activity of light-activated cyclases indirectly by the activation of CNG channels^21^,^22^,^32^. Olfactory (OLF)/T537S is a bovine CNG channel (CNGA2; ref. 34) which is highly sensitive to cAMP (*K*_0.5_∼14 μM) and even more to cGMP (K_0.5_∼0.7 μM). We co-expressed BeCyclOp::YFP and the OLF channel in oocytes, and used the two-electrode voltage-clamp technique to monitor light-induced cGMP production via cGMP-activated currents (Supplementary Fig. 5A). Indeed, a brief green laser flash (100 ms, 532 nm) induced∼5 μA of inward current (at −60 mV) in oocytes, 3 dpi of 0.6 ng BeCyclOp::YFP and 6 ng OLF cRNA each (Supplementary Fig. 5B). Light-induced currents became obvious already 100 ms after illumination. As shown by our *in vitro* assay, the photocycle of BeCyclOp is relatively fast with a decay time of 320 ms for the light-activated guanylyl cyclase activity (Fig. 2c); thus the photocurrent starts decreasing ∼1 s after activation, presumably due to cGMP diffusion and PDE activity (Supplementary Fig. 5C).

### BeCyclOp is a more effective GC than bPGC (BlgC) and EROS

A soluble photoactivated guanylyl cyclase (bPGC or BlgC) was generated from bPAC by three point mutations^23^, but only very recently (during revision of this manuscript) tested in animal cells^25^. To compare BeCyclOp::YFP and bPGC::YFP, we expressed them in oocytes (Supplementary Fig. 6A), and measured the cGMP concentrations obtained 3 dpi, in the dark, or after 2 min of illumination with blue light (464 nm, 10 μW mm^−^^2^; Supplementary Fig. 6B). These illumination conditions favoured bPGC with its action spectrum maximum in the blue and its higher light sensitivity. cGMP concentrations of bPGC-expressing oocytes, kept in dark, and those of control (that is, non-expressing) oocytes were similar. However, blue light yielded >50 times more cGMP in BeCyclOp-expressing oocytes (*N*=3) than in bPGC-expressing oocytes. Further, when illuminating for 20 min, bPGC-expressing oocytes contained significantly more cAMP than controls, while BeCyclOp-expressing oocytes did not exceed the cAMP level of controls (Supplementary Fig. 6C).

### Function of BeCyclOp in muscle cells of live *C. elegans*

**Figure 4:**
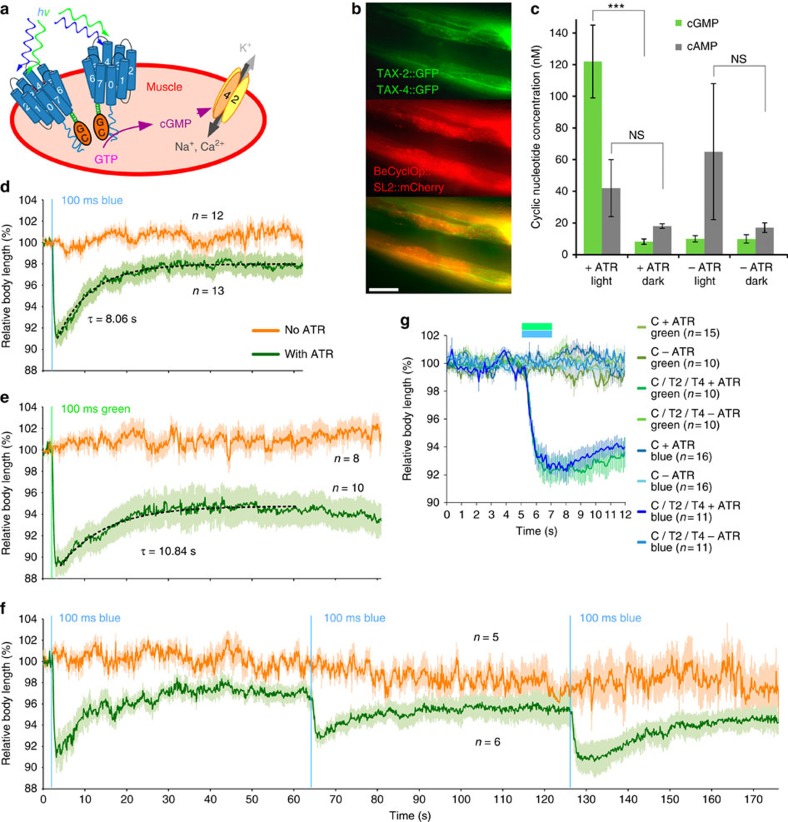
Light-induced muscle activation in *C. elegans* via BeCyclOp and a CNG channel. (**a**) Co-expression of BeCyclOp and the *C. elegans* CNG channel consisting of TAX-2 and TAX-4 subunits (not normally expressed in muscle; indicated by yellow and orange ovals) generates a light-activated system for cell depolarization and muscle activation. (**b**) Fluorescent micrographs of the head region of an animal expressing BeCyclOp from a bicistronic construct with mCherry, in body wall muscles, and co-expressing TAX-2::GFP/TAX-4::GFP. Anterior is to the left. Scale bar, 20 μm. Membrane expression of TAX-2/4 is clearly visible as well as cytosolic mCherry. (**c**) *In vivo* cGMP and cAMP concentration assessed in crude extracts derived from whole animals expressing BeCyclOp in muscle cells. The animals were cultivated in the absence or presence of all-trans retinal (ATR) and were illuminated with green light (540–580 nm, 150 μW mm^−2^, 15 min; L) or were kept in the dark (**d**) during extract preparation. *N*=6 experiments; Error bars, s.d. Statistically significant differences: one-way ANOVA (***P<0.001). (**d**–**f**) Body length measurements of animals before and following 100 ms light pulses of blue (450–490 nm, 0.9 mW mm^−2^; **d**,**f**) or green light (520–550 nm, 0.9 mW mm^−2^; **e**). Animals were raised in the absence or presence of ATR (orange or green curves, respectively). Only animals supplemented with ATR exhibit contractions. These are long lasting (mono-exponential fits are shown by dashed lines and the relaxation time constants are indicated), and can be repeatedly evoked (**e**). Mean normalized body length of the indicated number of animals; error bars, s.e.m. (**g**) Body length changes were induced only when BeCyclOp (‘C') was co-expressed with TAX-2/TAX-4 (‘T2/T4') and animals were raised in the presence of ATR. Illumination was either with blue light (blue graphs), or with green light (green graphs). Bars indicate 2 s illumination period.

Guanylyl cyclase function is abundantly found in sensory neurons in many animals. One widely used animal model in the neurosciences is *C. elegans*. To assess BeCyclOp's utility in animal optogenetics, we expressed a synthetic cDNA, codon optimized for *C. elegans*, in body wall muscle cells (BWMs), along with an intrinsic cGMP-gated heteromeric CNG channel found in many *C. elegans* sensory neurons, consisting of TAX-2 and TAX-4 subunits (Fig. 4a). TAX-2/4 form an unspecific cation channel both *in vitro*^35^ and *in vivo*^36^. Activation of this channel is expected to cause muscle depolarization and contraction. As BWMs are large, they can evoke macroscopic contraction of the animal. Cultivation of animals expressing BeCyclOp/TAX-2::GFP/TAX-4::GFP in BWMs (Fig. 4b) in the presence of ATR, and 100 ms illumination with blue (450–490 nm) or green (520–550 nm) light caused longitudinal body contractions of about 9–10% of the initial length within 1–1.5 s (rise time constant ∼0.36–0.38 s (*n*=10–13 animals); Fig. 4d,e). This was comparable to effects evoked by ChR2 expression and photostimulation in the same tissue^18^,^37^,^38^. As a control, we raised animals without ATR. Since the chromophore is absent in *C. elegans*, opsin optogenetic tools are expressed but non-functional in such animals. Also for BeCyclOp/TAX-2/4, no contraction could be observed without ATR addition. Light evoked a ∼12-fold increase in cGMP concentration, as determined from crude whole-animal extracts, compared with the dark condition (*N*=6; Fig. 4c). No evidence for BeCyclOp-mediated cAMP generation was found (*N*=4), although illuminated samples showed a trend to increased cAMP. This was independent of ATR (and thus, functional BeCyclOp), and may be due to a *C. elegans*-specific blue-light avoidance response^39^. Contractions evoked by photoactivated BeCyclOp/TAX-2/4 (with ATR) were long lasting and recovered to a plateau of about 3% within ∼30 s (*τ*_off_∼8.1 s (*n*=13 animals); blue light); for green light, the plateau was about 6% contraction (*τ*_off_∼10.8 s, *n*=10 animals). This indicates more pronounced cGMP generation when BeCyclOp is stimulated at its action spectral peak (Fig. 4e). In comparison, contractions evoked by ChR2 recover within 1–1.5 s (*τ*_off_∼0.58 s; ref. 38). Thus, in muscle, cGMP levels may be persistently increased by BeCyclOp activation, likely due to low PDE activity. Also, the TAX-2/4 channel, while it may partially desensitize, appears to not inactivate upon prolonged cGMP increase. Light-evoked contractions were repeatable: three consecutive light pulses over 3 min evoked comparable effects (Fig. 4f). Contractions more or less reached similar values, showing that BeCyclOp can be repetitively activated. No light-induced contractions were observed when BeCyclOp alone was expressed in BWMs, that is, without the TAX-2/4 channel (Fig. 4g). We could not measure photocurrents by electrophysiology, likely due to persistent cGMP and TAX-2/4 currents following animal dissection in white light.

### Light sensitivity of BeCyclOp in *C. elegans*

**Figure 5:**
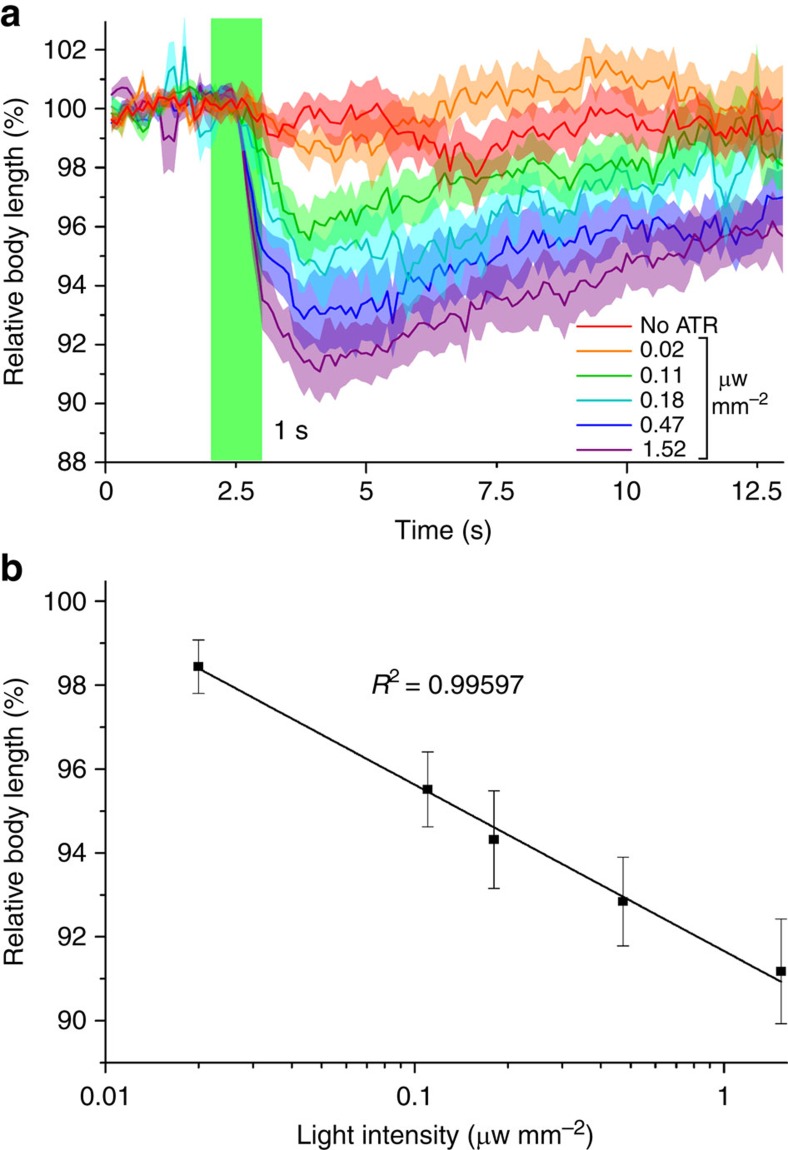
Light dose-response correlation of BeCyclOp-evoked body contractions in *C. elegans*. (**a**) Mean normalized body length chronograms of animals before, during and after 1 s green light stimuli (green bar) of the indicated intensities. The negative control −ATR was performed at 1.52 μW mm^−^^2^ (*n*=10 each, error bars, s.e.m.). (**b**) Dose-response graph (linear fit of contraction and the log of the light intensities used) shows a negative correlation. Above 1.52 μW mm^−^^2^, contraction reaches saturation.

We used the behavioural effects of BeCyclOp/TAX-2/4 stimulation to determine the light sensitivity in *C. elegans.* This indirect measure depends on BeCyclOp and TAX-2/4 expression levels, duration and wavelength of the light pulse, on- (and off-) rate of BeCyclOp's guanylyl cyclase activity, photocycle kinetics and expression of PDEs in BWMs. Illumination for 100 ms was sufficient to evoke contractions. Shorter light pulses also had effects, but these were less robust and variable. To determine the light dose required to evoke full effects, we used 1 s, 530 nm light pulses, at intensities between 0.02–1.52 μW mm^−^^2^ (*n*=10 animals each; Fig. 5a). The peak contractions showed a negative linear correlation with the log of the light intensity (Fig. 5b). Illumination of animals raised without ATR (1.52 μW mm^−^^2^) evoked no contraction. The light intensity required to evoke robust contraction is ∼200-fold less than what is typically needed to evoke muscle contractions using ChR2 (refs 18, 38), thus BeCyclOp appears to be much more light sensitive; however, this may be an apparently higher light sensitivity, as BeCyclOp/TAX-2/4 uses second messenger amplification, and a high-conductance channel, unlike ChR2 photocurrents that need to trigger muscle contraction directly.

### Activating BeCyclOp in O_2_ sensors slows down locomotion

**Figure 6:**
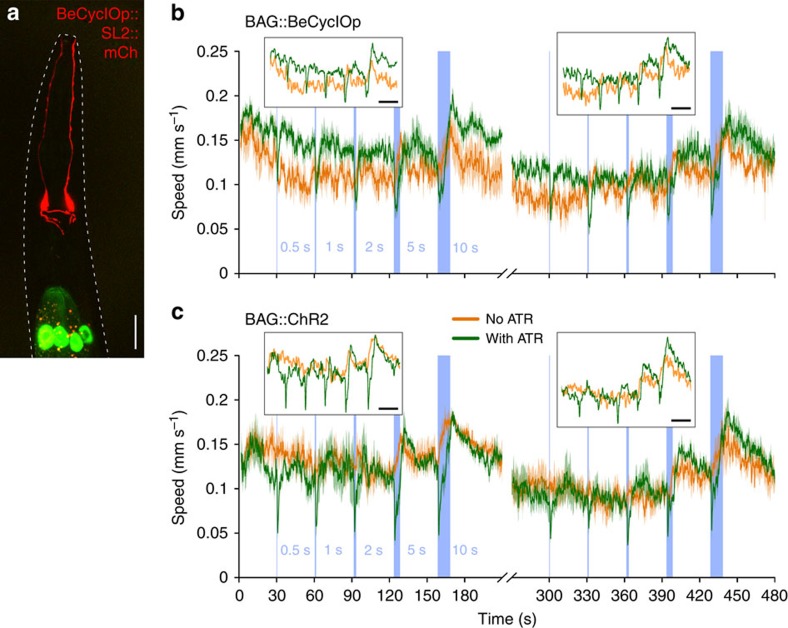
BeCyclOp triggers O_2_ sensory BAG neurons that intrinsically use cGMP signalling. (**a**) Confocal image of expression of BeCyclOp::SL2::mCherry bicistronic construct in the BAG neuron pair in the head of *C. elegans*. Cell bodies, nerve ring processes and sensory dendrites reaching the nose are visible. Outline of the animal indicated by a dashed white line, anterior is up. Green fluorescence: GFP co-expression marker localized to intestinal cell nuclei. Scale bar, 20 μm. (**b**) Mean absolute locomotion speed of the indicated numbers of animals expressing BeCyclOp in BAG neurons, during repeated blue light exposure (470 nm, 70 μW mm^−^^2^) for the indicated periods (blue bars), with 30 s interstimulus intervals, as deduced from video analysis using a multiworm tracker. Animals in the absence (orange curves) or presence of ATR (green curves) in the culture media are compared. Speed increase in animals without ATR is due to photophobic behavior. *N*=5 (4 for no ATR) experiments with *n*=15–20 animals each; error bars, s.e.m. (**c**) As in **b**, but animals expressing ChR2 in BAG neurons were used instead. *N*=3 experiments with *n*=15–20 animals each; error bars, s.e.m. The insets show mean speeds, as a moving average, using a sliding window bin corresponding to 1 s for filtering the data. Black bars, 30 s.

Cell body in anterior ganglion (BAG) neurons of *C. elegans* are O_2_/CO_2_ sensors that detect a drop in O_2_ as well as increases in CO_2_ and evoke a transient slowing response^40^,^41^,^42^,^43^. To detect O_2_, BAG neurons use O_2_-binding soluble receptor guanylyl cyclases, GCY-31 and GCY-33, as well as the TAX-2/4 channel. We expressed BeCyclOp in the BAG neurons using the *flp-17* promoter (Fig. 6a; ref. 42). Locomotion speed was measured using a multiworm tracker^44^, equipped with a ring of blue light-emitting diodes, thus enabling to apply light of sufficient intensity to a whole-culture dish. Photostimulation of BeCyclOp in BAG caused transient slowing responses (Fig. 6b; Supplementary Movie 1). Stimuli of increasing duration (500 ms, 1, 2, 5 and 10 s; 30 s intervals; *N*=5 (4 for no ATR) experiments; *n*=15–20 animals each) evoked significant slowing responses of increasing duration, though there was always a transient minimum, following which the animals gradually increased their speed again, despite ongoing light stimulation (see also insets in Fig. 6, as well as Supplementary Fig. 7 for analysis of speed changes within 1 s before and during the light stimuli). These behaviours were similarly observed for animals expressing ChR2 in BAG (*N*=3 experiments; *n*=15–20 animals each; Fig. 6c; ref. 42). For both optogenetic tools, stimulus protocols could be repeated, leading to similar results. In control experiments with animals raised without ATR, light stimuli >2 s evoked a transient increase in locomotion speed. This is attributable to the function of the *C. elegans* ultraviolet/blue-light sensor, LITE-1, which evoked escape behavior^39^. Light-evoked slowing responses due to BAG activation, both in response to BeCyclOp or ChR2 activation, apparently overcame light-evoked photophobic responses. When the prolonged blue-light stimuli ended, animals showed a rebound speed increase that was more pronounced for longer stimuli; locomotion speed then fell back to initial speeds within ∼20–30 s. In sum, BeCyclOp enables triggering cGMP signalling in animals, either in sensory cells using cGMP, or in other cells ectopically expressing CNG ion channels.

## Discussion

Here we molecularly characterized a new class of type I opsins, the cyclase opsins (CyclOps) from several fungal species, for photoactivation, action spectrum and light sensitivity, after heterologous expression, in part with CNG channels. We show enzymatic specificity, high turnover, virtually non-existing dark activity and unusual topology of *Blastocladiella* BeCyclOp. Compared with other CyclOps, BeCyclOp was most effective. To assess its potential in animal optogenetics, we expressed it in *C. elegans* muscles, together with the TAX-2/4 CNG channel, as well as in oxygen sensory neurons that intrinsically use cGMP for signalling. Both cell types could be photoactivated, and macroscopic behaviours were rapidly induced and turned off, or were long-lasting, depending on the tissue. BeCyclOp thus qualifies as a new optogenetic tool for specific cGMP manipulation in live animals.

Microbial rhodopsins attracted much attention in recent years because of their impact in optogenetics. The first type I rhodopsin described was bacteriorhodopsin^12^, a light-activated H^+^ pump. Bacteriorhodopsin resembles animal rhodopsins in the common seven transmembrane structure and in its Schiff-base binding of the retinal chromophore to a lysine in transmembrane helix 7. This overall structure is preserved in all subsequently discovered microbial rhodopsins (Cl^−^ pumps, the phototaxis sensory rhodopsins, the channelrhodopsins and so on), and type I rhodopsins share homology in transmembrane helices 3 and 7, and in amino acids of the retinal-binding pocket. Fungal CyclOps now add a novel aspect to the diversity of type I rhodopsins by the way of their additional N-terminal transmembrane helix 0 (Supplementary Fig. 2). Based on the absent signal peptide and transmembrane helix prediction, the N terminus was expected to be cytosolic. We confirmed this by our BiFC experiment. To our knowledge, this is the first finding of an N terminus of any type I or type II rhodopsin being intracellular. Compared with the full-length BeCyclOp, the N-terminally shortened CyclOpS showed higher dark activity and lower light-induced cGMP production, suggesting that the N terminus is involved in tight light regulation of the cytosolic guanylyl cyclase activity.

We show by expression in *Xenopus* oocytes and in muscle and nerve cells of *C. elegans* that BeCyclOp ‘behaves' well and that its activity is under tight light control. Other fungal CyclOps we tested were much less efficient, and/or less specific. The preparation of CyclOp-containing membranes from oocytes enabled biophysical characterization of these proteins without interference by PDEs. The very high ratio of light versus dark activity (L/D) of 5,000 observed for BeCyclOp was reduced to 1,100 by fusing YFP to the C terminus, mainly due to a fourfold increased dark activity. Previously the highest L/D ratio (300) was reported for bPAC from *Beggiatoa*^22^.

The action spectrum of BeCyclOp is a typically broad rhodopsin spectrum with a 530 nm maximum. Though BeCyclOp is most sensitive to green light, it still responds to violet and red light; the latter may further facilitate its use in animals, deep within tissue. To avoid pre-activation, we worked under far-red light (650–720 nm) for experiments with BeCyclOp membranes, as well as with transgenic *C. elegans*, as activity here was undistinguishable from dark activity. The time constant for light-activation of the guanylyl cyclase is expected to be faster than 1 ms if BeCyclOp is similar to other type I opsins; in fact it is too fast to be measured by our methods. Also inactivation upon light-off (τ∼320 ms) is relatively fast for BeCyclOp, contrasting previously described flavoproteins and ACs bPAC and mPAC (*τ*∼20 and 10 s, respectively^22^,^32^). Provided PDEs are expressed in the cells addressed by BeCyclOp, this enables not only good optogenetic ON, but also OFF control. The faster photocycle of BeCyclOp resulted in light sensitivity lower than that of bPAC and mPAC, but even more light-sensitive mutants may be obtainable. The only PGC available until now is the mutated PAC from *Beggiatoa* (termed bPAC^22^, or BlaC^23^), which was first engineered by Ryu *et al*.^23^, to a PGC and named BlgC. This PGC was recently renamed EROS, and shown to mediate light-inducible penile erection in rats^25^. Here we mutated a human codon-optimized bPAC cDNA^22^ by three mutations, just as Ryu *et al*.^23^, to convert it to bPGC (Supplementary Fig. 6), identical in aa sequence to BlgC and EROS. bPGC is much less active than BeCyclOp, with 50-fold lower cGMP production at bPGC-favouring illumination conditions (dim blue light). Furthermore, BeCyclOp is highly specific for cGMP production, since we detected no cAMP generation, while bPGC produced at least one cAMP for every four cGMP molecules under *in vivo* conditions (oocyte, Supplementary Fig. 6), or one cAMP per seven cGMP under ideal *in vitro* conditions^23^. Our *in vitr*o assay for BeCyclOp PGC activity yielded ∼17 cGMP s^−1^ at 20 °C, the highest reported turnover for any characterized light-activated nucleotidyl cyclase to date. For the cytosolic bPGC (aka BlgC or EROS), we estimate a turnover of 0.2 cGMP s^−1^ as its activity seems 100-fold lower than that of BeCyclOp or of bPAC. Summarizing the advantages of BeCyclOp, we found that it is highly active and specific for cGMP production, and it has the highest so far reported dynamic range for any photoactivated nucleotidyl cyclase with a very high ratio of light versus dark activity (L/D) of 5,000.

BeCyclOp is highly useful for animal optogenetics. It generated cGMP in *C. elegans* muscle cells, which could be photo-depolarized when the TAX-2/4 CNG channel was co-expressed. This combination, due to the built-in signal amplification through second messenger generation, may even be used as a new optogenetic tool for efficient cellular depolarization with low light, though the need for co-expression of three genes (or two, if the OLF channel is used) makes this less versatile than ChR2. In the absence of PDEs in the respective cell type, the signal turns off slowly; thus, combination with the recently described red-light-activated PDE (LAPD) may be beneficial^45^.

However, BeCyclOp is most useful as an optogenetic tool in cells that intrinsically signal via cGMP, and is of particular interest for functional analysis of sensory neurons. In the *C. elegans* BAG neurons that sense ambient O_2_ and CO_2_, photostimulation of ChR2 led to a transient decrease in locomotion speed (ref. 42, and Fig. 6c), and the same effects were observed in animals in which we expressed and stimulated BeCyclOp in BAG neurons. Unlike ChR2, which merely depolarizes the BAG neurons, BeCyclOp evokes this outcome by triggering the cGMP second messenger upstream of the intrinsic CNG channel TAX-2/4. BAG neurons may use the intermediate cGMP signal also for modulation of the sensory output, or for plasticity. For both BeCyclOp and ChR2, prolonged BAG stimulation led to slowing of locomotion speed. However, the slowing was transient, despite ongoing light stimulus. This may represent desensitization or even habituation. Using ChR2, it is indistinguishable if this desensitization is evoked in BAG, possibly involving modulation of cGMP signalling, whether it is affected at BAG output synapses, or even in downstream neuronal networks that cause and execute slowing. Using BeCyclOp, we can now likely exclude that prolonged stimulation of BAG leads to modulation of intrinsic cGMP signalling and that the desensitization response must occur downstream of BAG depolarization.

BeCyclOp will permit detailed studies of sensory neuron function in *C. elegans* and other animals. In *C. elegans*, it is particularly useful to study environmental cues like odorants, tastants, gases or temperature, and how cGMP signalling may shape the response of the respective neurons and the behavioural consequences of their activation. But BeCyclOp will prove useful also in other systems where cGMP signalling plays a role, for example, in sperm swimming, erectile dysfunction, NO signalling and smooth muscle relaxation, or even in animal vision, where cGMP is degraded upon illumination. For the latter, BeCyclOp would have to be combined with a PDE; conversely, BeCyclOp may become an important optogenetic tool to model (that is, ‘acutely' induce) retinal degeneration as observed in PDE6 mutants.

## Methods

### *Xenopus* oocyte expression

The five fungal CyclOp sequences, including BeCyclOp (=BeGC1) were synthesized by GeneArt Strings DNA Fragments (Life technologies, Thermo Fisher Scientific) according to the published aa sequence^26^, with codon usage optimized to *Mus musculus*. The DNA was inserted into the oocyte expression vector pGEMHE within N-terminal *Bam*HI and C-terminal *Xho*I restriction sites; YFP was attached to its C-terminal end. The CyclOp BiFC construct was made by ligating the C-terminal 85 aa of YFP (aa 155–239; YC) to the N terminus of BeCyclOp. The N-terminal 155 aa of YFP (YN) where present at the BeCyclOp C terminus in the BeCyclOp::YFP construct and shortened by a present *Kpn*I restriction site. The two fragments for ligation were amplified from BeCylOp::YFP with primers BeCyclOpKpn5F (5′-CGGGGTACCATGAAGGACAAGGACAACAAC-3′) and YFPN155HdR (5′-GCCCAAGCTTAGGCCATGATATAGACGTTGT-3′) for BeCyclOp::YN and primers YFPC85BHF (5′-CGGGATCCGCCACCATGGCCGACAAGCAGAAGAACGG-3′) and YFPendKpnR (5′-CGGGGTACCAACTTCATTTTCATAGCAAAATCTAG-3′) for YC. The two fragments were then inserted between *Bam*HI and *Xho*I restriction sites. The bPGC was a triple mutant of bPAC (K197E/D265K/T267G). The YFP was attached directly to its C-terminal to make bPGC::YFP. The sequence was confirmed by sequencing. NheI-linearized plasmid DNA was used for the *in vitro* generation of cRNA with the AmpliCap-MaxT7 High Yield Message Maker Kit (Epicentre Biotechnologies).

### HEK293T cell expression

The BeCyclOp cDNA fragment was digested with *Bam*HI and *Hin*dIII restriction enzymes and then ligated into pBK-CMV vector between the *Bam*HI and *Hin*dIII restriction sites for HEK293T cell transfection.

### *C. elegans* molecular biology

The following plasmids were constructed: **pJN55** [pmyo-3::tax-2::GFP]: A 2415, bp long pmyo-3 fragment was amplified using primers oJN144 (5′-AATGAAATAAGCTTGCGGCTATAATAAGTTCTTGAA-3′) and oJN145 (5′-TGATACATATCCCGGTCTAGATGGATCTAGTGGTC-3′). This was ligated into pgcy-8::tax-2::GFP (pBY3117 in ref. 46), digested with *Sph*I and *Xma*I (resulting in a 6,790 bp long DNA fragment) using 'in-fusion cloning' (Clontech). For **pJN58** [pmyo-3::tax-4::GFP], the strategy was as for pJN55, but taking a 6,589 bp long fragment obtained upon digestion of pgcy-8::tax-4::GFP (pBY2974 in ref. 46) with *Sph*I and *Xma*I was used as the vector backbone. We also removed a mutation, T724A, which we found in the parent plasmid pBY2974, using the Q5 site-directed mutagenesis (New England Biolabs Inc.) kit and primers oJN154 (5′-TGATCTACCAACGGGAACTGA-3′) and oJN155 (5′-ATTGTTTTTGTTTGTCGGAGAC-3′). For **pJN63** [pmyo-3::BeCyclOp::SL2::mCherry], cDNA encoding BeGC1 (*B. emersonii* rhodopsin/guanylyl cyclase 1, GenBank: KF309499.1; equivalent of BeCyclOp) was codon optimized for expression in *C. elegans* using the web-tool: http://worm-srv3.mpi-cbg.de/codons/cgi-bin/optimize.py^47^. Three artificial introns were added to the sequence for mRNA stabilization. The resulting gene was synthesized by Eurofins Genomics (Ebersberg, Germany) in a pUC57 vector. BeCyclOp was sub-cloned into pmyo-3::SL2::mCherry vector backbone using *Kpn*I and *Xba*I. For **pJN66** [pflp-17::BeCyclOp::SL2::mCherry], the 3.3 kb of *pflp-17* (ref. 42) was amplified from genomic DNA using primers oJN195 (5′-CCTTTTGCTCACATGCCTTGAAGCTTTTCCTCTG-3′) and oJN196 (5′-GTCCTTCATTCTAGACTGGAAAAATAAAGTTTTGCG-3′). The PCR fragment was cloned into pmyo-3::BeCyclOp::SL2::mCherry following digestion with *Xba*I and *Pci*I, thus replacing *pmyo-3* with *pflp-17* using 'in-fusion' cloning.

### *C. elegans* strains

Cultivation was on nematode growth medium (NGM) in the presence of the *Escherichia coli* strain OP50-1 according to standard methods^48^. The following strains were used or generated: **CX10293:** *kyEx2416 [pflp-17::ChR2::mCherry; unc-122::gfp]*^42^; **ZX1743:** *lite-1(ce314); zxEx851[pmyo-3::BeCyclOp::SL2::mCherry; pmyo-3::tax-2::GFP; pmyo-3::tax-4::GFP]*, **ZX1745:** *zxEx852[pflp-17::BeCyclOp::SL2::mCherry; pelt-2::GFP-nls]*; **ZX1746**: *lite-1(ce314); zxEx853*[*pmyo-3::BeCyclOp::SL2::mCherry*]. Transgenic *C. elegans* were obtained by microinjection of DNA into the gonads of nematodes by standard procedures^49^. For ZX1743/*zxEx851*, 15 ng μl^−1^ pmyo-3::BeCyclOp::SL2::mCherry; 5 ng μl^−1^ pmyo-3::tax-2::GFP; and 5 ng μl^−1^ pmyo-3::tax-4::GFP] were microinjected into *lite-1(ce314)* background. For ZX1745/*zxEx852*, 1.5 ng μl^−1^ pflp-17::BeCyclOp::SL2::mCherry was microinjected into N2 worms to obtain the transgenic line. For ZX1746/*zxEx853*, 15 ng μl^−1^ pmyo-3::BeCyclOp::SL2::mCherry was microinjected into *lite-1(ce314)* background.

### Oocyte extract preparation

For expression in oocytes and extraction for the cGMP/cAMP assay, cRNAs (∼30 ng) from different CyclOps were injected into *Xenopus* oocytes, and were allowed to be expressed for 3 days. Four to six oocytes were pooled as one sample and homogenized (in dark or after illumination) in 0.1 N HCl by pipetting using a 10–100 μl Eppendorf Pipette. The supernatant was centrifuged at 12,000 r.p.m. for 6 min and then used for the cGMP (or cAMP) assay with DetectX High Sensitivity Direct cGMP (or cAMP) Chemiluminescent Immunoassay Kit (Arbor assays). For *Xenopus* oocyte membrane extraction and *in vitro* assay, 20 oocytes were washed three times with solution A (83 mM NaCl, 2 mM MgCl_2_, 1 × Protease Inhibitor Cocktail (Roche) and 10 mM HEPES, pH 7.5) and then homogenized in 1 ml of solution A on ice. The yolk and cellular debris were sedimented at 800*g* at 4 °C for 20 min, and the pellet was discarded. The supernatant was transferred to a fresh tube and the membrane fraction was then sedimented at 20,000*g* at 4 °C for 20 min. The membrane pellets were gently washed twice with 800 μl solution A and resuspended in 80 μl solution A. Membrane extract (2 μl) was mixed with 18 μl Guanylate Cyclase reaction buffer mix (75 mM Tris/HCl, pH 7.5, 10 mM MgCl_2_, 5 mM dithiothreitol, 1 mM ATP, 2 mM GTP) for one reaction. 180 μl Sample Diluent (containing 0.1 M HCl) was used to stop the reaction for the cGMP quantification assay. The cGMP (or cAMP) assay was performed with DetectX High Sensitivity Direct Cyclic GMP (or cAMP) Chemiluminescent Immunoassay Kit (Arbor assays).

### Quantification by fluorescence

To quantitate BeCyclOp expression, we used the fluorescence emission to quantitate the YFP-tagged protein amount in our samples. Purified YFP protein standard (Evrogen JSC; 1 mg ml^−1^), was added to PBS in buffer, that is, 0, 5, 10, 20, 40, 80, and 160 ng, in 96-well plates with three repeats. Fluorescence emission was then measured at 538 nm using a Fluoroskan Ascent microplate fluorometer with 485 nm excitation. *Xenopus* oocyte membranes were isolated the same way as used for the *in vitro* guanylyl cyclase assay. The measured emission value was then fitted to the previously measured YFP standard to determine the YFP amount in the sample. To calculate the BeCyclOp turnover, the guanylyl cyclase activity was measured immediately by the *in vitro* assay.

### HEK293T extracts

For expression and extraction of BeCyclOp, HEK293T cells were transfected with BeCyclOp in pBK-CMV vector using calcium–phosphate transfection. To this end, cells were seeded in a six-well plate to 80% confluency (800,000 cells) in 2 ml of cell culture medium (DMEM+10% FCS) and transfected with 2 μg of plasmid mixed with 500 μl of transfection buffer (250 mM CaCl_2_, 25 mM HEPES pH 7.05, 70 mM NaCl, 0.75 mM Na_2_HPO_4_, 25 μM Chloroquine). Cell culture medium was exchanged after overnight incubation and retinal was added to 1 μM to some samples. One half of the samples was exposed for 15 min to blue light in the cell culture incubator. Cells were harvested 36 h post transfection (estimated efficiency of transfection was 50%), homogenized and solubilized 1:5 in 0.1 N HCl. The supernatant after centrifugation was used for the cGMP (or cAMP) assay with DetectX High Sensitivity Direct cGMP (or cAMP) Chemiluminescent Immunoassay Kits (Arbor assays).

### *C. elegans* extract preparation

For *C. elegans in vivo* cGMP or cAMP assays, 30 L4 larvae (strain ZX1746, worms expressing BeCyclOp only in BWMs) were picked onto NGM plates supplemented with OP50-1 with or without 100 μM ATR each. The plates were kept in a 20 °C incubator for 3 days. Afterwards, the animals were rinsed off the plates with M9 buffer, subsequently washed with M9 buffer 4–5 times to remove bacteria and transferred into 24-well plates. The animals were illuminated with green light (540–580 nm, 150 μW mm^−2^) using a Leica fluorescence stereomicroscope for 15 min. Following illumination, the M9 buffer was substituted by 0.1 M HCl (1:10 dilution to the worm pellet) and the animals were subjected to three freeze-thaw cycles with liquid nitrogen. Afterwards, the animals were vortexed with 0.25–0.5 mm glass beads in 0.1 M HCl for 1 min. The supernatant after centrifugation (2,000 r.p.m., 1 min) was used for the cGMP (or cAMP) assay performed with DetectX High Sensitivity Direct cGMP (or cAMP) Chemiluminescent Immunoassay Kit (Arbor assays).

### BeCyclOp action spectrum

Light of different wavelengths was obtained by narrow bandwidth interference filters (Edmund Optics) together with a PhotoFluor II light source (89North). The wavelength was further confirmed with a spectrometer (Ocean Optics). The light intensities at different wavelengths were all adjusted to ∼0.02 mW mm^−^^2^ and measured with a Laser Check photometer (Coherent Technologies). The action spectrum was normalized to the activity at 541 nm.

### Oocyte electrophysiology

Cells were injected with the indicated amount of cRNA mixture of BeCyclOp and the OLF/T537S channel. Oocytes were incubated in ND96 solution (96 mM NaCl, 2 mM KCl, 1 mM MgCl_2_, 1 mM CaCl_2_, 10 mM HEPES and 50 μg ml^−1^ Gentamycin, pH 7.4) containing 1 μM ATR. Two-electrode voltage-clamp recordings of photocurrents were made in Ringer's solution (110 mM NaCl, 5 mM KCl, 2 mM BaCl_2_, 1 mM MgCl_2_, 5 mM HEPES, pH 7.6) at a holding potential of −100 mV. A 532 nm laser was used as light source.

### Fluorescence microscopy

YFP fluorescence imaging of oocytes was done using a confocal laser scanning microscope (LSM 5 Pascal, Carl Zeiss) equipped with a Zeiss Plan-Neofluar 10 × /0.5 objective. Images were processed using LSM 5 Image Browser and exported for insertion into figures. Expression of TAX-2::GFP or TAX-4::GFP and BeCyclOp::SL2::mCherry in BWMs of *C. elegans* was analysed on an Axio Observer.Z1 (Zeiss) microscope equipped with a GFP and Rhodamine-specific excitation/emission filter set, respectively. Images were obtained with a Hamamatsu ORCA-Flash 4 sCMOS camera and Micro-manager software. A confocal laser scanning microscope (Leica SP5) was used to image BeCyclOp::SL2::mCherry expression in BAG neurons using 488 and 561 nm lasers.

### *C. elegans* behavioural assays

Transgenic strains were kept in dark on standard NGM plates (5.5 cm diameter; 8 ml NGM) with OP50-1 bacteria without or with ATR at 20 °C. Plates containing ATR were prepared by spreading 200 μl of OP50-1 culture containing 100 μM of ATR (diluted in ethanol). For the contraction assay, transgenic L2–L3 larvae were selected for muscle fluorescence under a Leica MZ16F dissection scope and transferred to freshly seeded ATR plates and thereafter kept in the dark. Measurements of contractions and elongations were performed as described previously^37^. L4 larvae (young adult animals for data in Fig. 4g) were individually placed on plain NGM plates and assayed on an Axio Scope.A1 microscope (Zeiss, Germany) with a 10 × objective and transmission light filtered through a red 675±50 nm bandpass filter. For colour illumination, the light of a 50 W HBO lamp was channelled through excitation bandpass filters of 470±20 nm (blue illumination) and 535±15 nm (green illumination). Both blue and green illumination had an intensity of 0.9 mW mm^−2^. For the intensity profile, 530±7.5 nm (green illumination) from a monochromator (Polychrom V) was used. Intensities were individually adjusted using the neutral density filters (Zeiss, Germany); intensity was measured using an S120UV Sensor with PM 100 power metre (Thorlabs, Dachau, Germany). Video recordings of worms were done using a CMOS camera (DCC1545M, Thorlabs, Dachau, Germany). Duration of illumination was defined by a computer-controlled shutter (Sutter Instruments, USA) using a custom written LabView script^50^,^51^. Consecutive frames were extracted from video micrographs of behaving worms for body length analysis using a custom written LabView script^50^,^51^. Measured length of individual animals was normalized by the mean length (averaged over 20 frames) before the photostimulation and followed over hundreds of consecutive movie frames (at 10 Hz). Length chronograms of multiple worms were then averaged. Displayed are means±s.e.m. For the speed assay, L4 stage animals were picked onto NGM agar plates supplemented with 100 μM of ATR. After 10–24 h of feeding in the dark, 15–20 young adults were transferred to an NGM plate and starved for 1 h. A multiworm tracker, which can analyse ∼100 animals simultaneously^44^ was used to follow behavior by video tracking. Blue light illumination was provided with a custom-made ring of high power 470 nm blue light-emitting diodes. The transmission light source was covered with a red film that cuts off all wavelengths below 590 nm. The NGM plate was covered with a lid, on top of which a neutral density filter was placed such that the blue light intensity incident on worms was 70 μW mm^−2^. Speed was computed using ‘Choreography', an additional programme for off-line analysis of different behavioural parameters^44^.

### Bioinformatics

Sequence alignments were done using ClustalX 2.1 (ref. 52) and coloured with BoxShade (http://www.ch.embnet.org/software/BOX_form.html). For the amino acid representation of type I rhodopsin transmembrane helix 3, a web-based tool was used (http://weblogo.berkeley.edu/)^53^. Transmembrane helix prediction was done using the TMHMM 2.0 web-based tool (http://www.cbs.dtu.dk/services/TMHMM/)^54^. The snake plot of BeCyclOp was done using Protter (http://wlab.ethz.ch/protter/start/)^55^, utilizing the Phobius algorithm^56^.

Sample size was chosen so that statistical significance could be achieved within the accuracy of the respective experiment.

## 

## Additional information

**How to cite this article:** Gao, S. *et al*. Optogenetic manipulation of cGMP in cells and animals by the tightly light-regulated guanylyl-cyclase opsin CyclOp. *Nat. Commun.* **6**:8046 doi: 10.1038/ncomms9046 (2015).

## Supplementary Material

Supplementary Information Supplementary Figures 1-7, Supplementary Table 1 and Supplementary References

Supplementary Movie 1 *C. elegans* expressing BeCyclOp in oxygen sensing BAG neurons. Animals were filmed before and during several pulses of green (530 nm) light, the speed of the video is increased 3 fold to emphasize the locomotion slowing effects.

## Figures and Tables

**Table 1:** BeCyclOp, AmCyclOps, and CaCyclOp membrane extract activity (cGMP and cAMP generation) under dark (D) and 0.5 mW mm^−2^ 532 nm green laser illumination (L).

**Membranes from oocytes expressing (nanograms of cRNA injected)**	**cGMP (pmol min**^**−1**^**)**			**cAMP (pmol min**^**−1**^**)**	
	**Dark**	**Green**	**L/D**	**Dark**	**Green**
Control oocytes (0)	NA	NA		NA	NA
AmCyclOp1 (28)	0.04±0.003	3.7±0.7	93	NA	NA
AmCyclOp2 (28)	NA	NA		NA	NA
AmCyclOp3 (28)	0.7±0.03	0.7±0.03	1	NA	NA
AmCyclOp1+3 (14+1.4)	0.09±0.006	3.3±0.04	37	NA	NA
AmCyclOp1+2 (14+14)	0.008±0.007	3.2±0.01	400	NA	NA
AmCyclOp2+3 (14+1.4)	0.02±0.001	0.02±0.02	1	NA	NA
AmCyclOp1+2+3 (7+7+1.4)	0.01±0.001	2.6±0.2	260	NA	NA
CaCyclOp (28)	1.2±0.1	280±2	230	NA	0.2±0.04
**Different** **oocyte batch**					
BeCyclOp no YFP (28)	0.2±0.1	1,000±150	5,000	NA	NA
BeCyclOp (28)	0.8±0.08	920±200	1,100	NA	NA

AmCyclOp, guanylyl cyclase rhodopsin from *Allomyces macrogynus*; BeCyclOp, guanylyl cyclase rhodopsin from *Blastocladiella emersoni*; CaCyclOp, guanylyl cyclase rhodopsin from *Catenaria anguillulae;* cRNA, coding RNA; L/D, light and dark ratio; NA, no obvious activity detectable.

cRNA injection amounts as indicated. Final activity refers to the activity of membrane extract from 1 oocyte. All constructs except ‘BeCyclOp no YFP' are with C-terminal YFP tag. *N*=3 experiments; errors: s.d.
