# Supplementary material for: Optogenetic manipulation of cGMP in cells and animals by the tightly light-regulated guanylyl-cyclase opsin CyclOp
Source: Nat Commun. 2015 Sep 8;6:8046. doi: 10.1038/ncomms9046 (PMC4569695; doi:10.1038/ncomms9046)
Supplement: Supplementary Information — Supplementary Figures 1-7, Supplementary Table 1 and Supplementary References [file ncomms9046-s1.pdf]

Supplementary Information

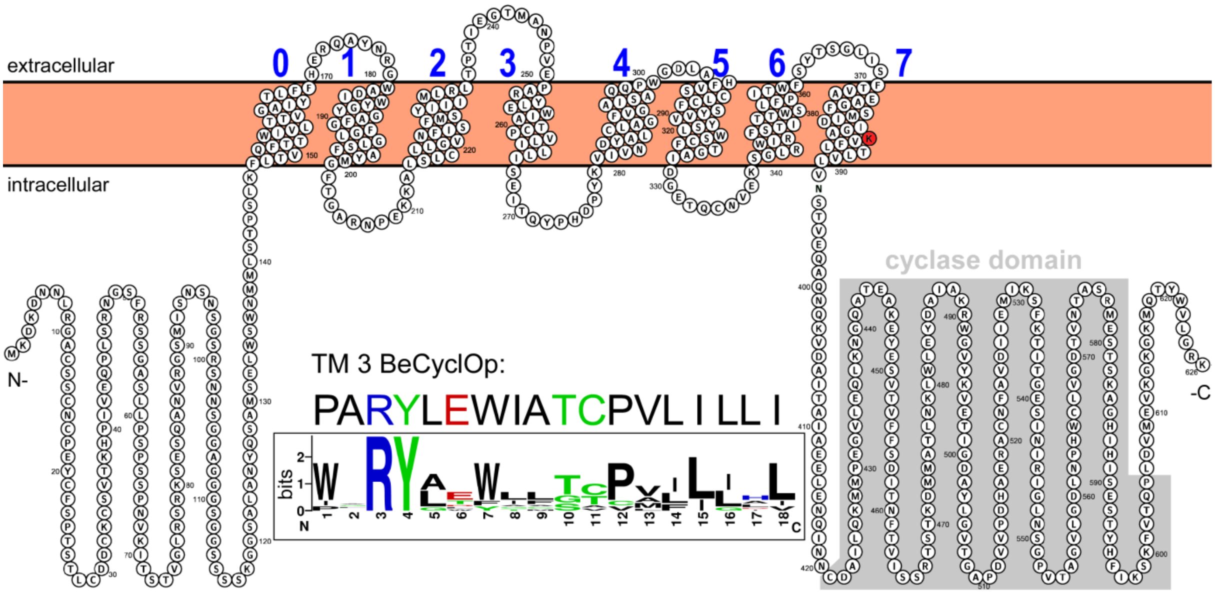

Supplementary Figure 1. Predicted structure of BeCyclOp

A) The BeCyclOp sequence. Transmembrane helices are predicted by TMHMM[1] in combination with sequence alignment. Picture drawn by Protter[2]. Numbering of TM helices based on the homology and topology of BeCyclOp TM helices and type I rhodopsin helices. The lysine residue in TM 7, to which retinal is bound by a Schiff base, is labeled red. The putative cyclase domain, with N- and C-terminal limits based on alignments with other type III guanylyl cyclases, is shaded in grey. Inset: The BeCyclOp 4th TM helix (TM 3) sequence and sequence logo of the highly conserved 3rd helix of microbial rhodopsins, based on the alignment of BeCyclOp, *H. salinarum* bacteriorhodopsin and halorhodopsin (HsBR and HsHR), *Natronomonas* halorhodopsin (NpHR), *Anabaena* sensory rhodopsin (ASR), *Natronomonas* sensory rhodopsin II (NpSRII), *Chlamydomonas* channelrhodopsins 1 and 2 (ChR1 and ChR2), and *Volvox* ChR1, was generated by Weblogo 2.8.2 (ref. 3).

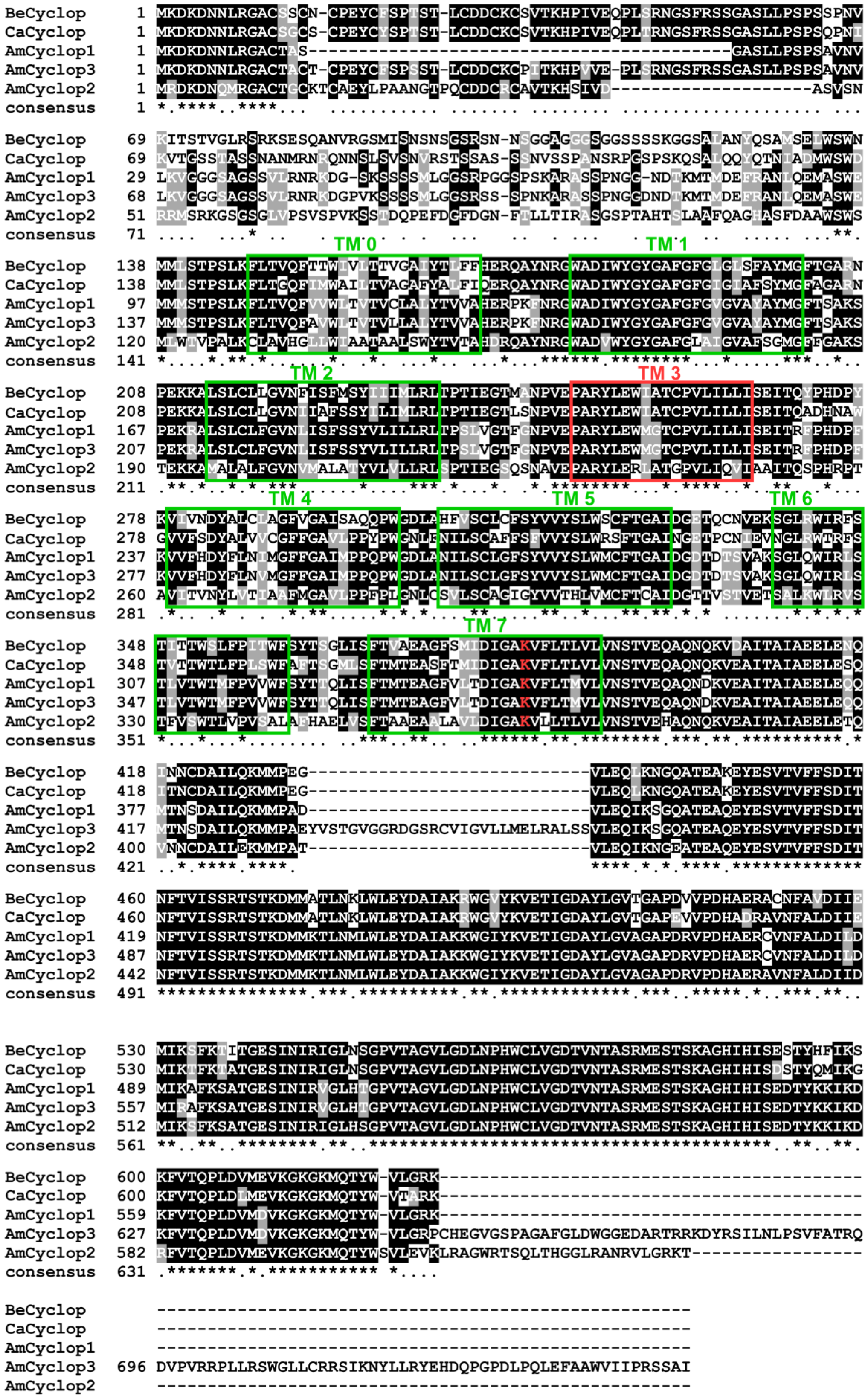

Supplementary Figure 2. Alignment of fungal CyclOps studied in this work.

Sequence alignment of the BeCyclOp, AmCyclOp1-3 and CaCyclOp sequences. The TM helices, as predicted in Fig. S1, are marked with a green box, the TM 3 with a red box. Black shading indicates identity and grey shading homology in >50% of the sequences. The consensus is shown too. The conserved lysine in TM 7, forming the retinal binding Schiff base, is printed in red.

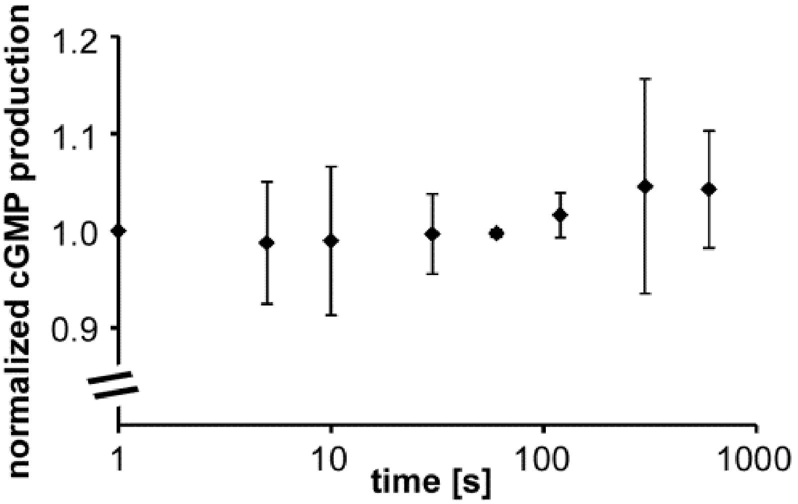

Supplementary Figure 3. Long term stability of cGMP generated by BeCyclOp in *Xenopus* membrane fractions after 1s stimulation.

For this in *vitro* assay with *Xenopus* oocytes membranes containing BeCyclOp, 1 s 0.4 mW mm^{-2} 532 nm green light was applied, and the cGMP concentration was measured after different resting periods for up to 10 minutes. n = 3, error bars = SD.

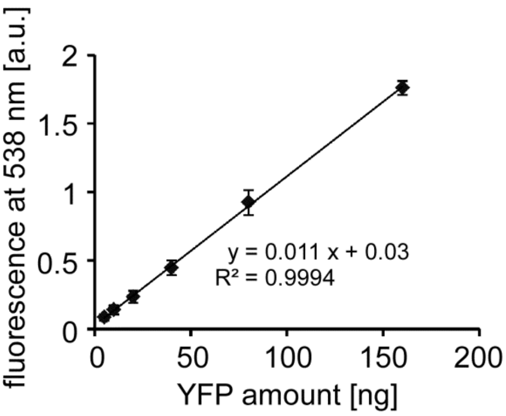

Samples	BeCyclOp amount (pmol / oocyte)
BeCyclOp	0.36 ± 0.03
CaCyclOp	0.21 ± 0.03
AmCyclOp1	0.38 ± 0.01

	total BeCyclOp [pmol]	BeCyclOp per reaction [pmol / 2 µL]	GC activity in light [pMol / (min * 2 µL)]	GC turnover / BeCyclOp molecule [cGMP molecules / s]
BeCyclOp #1	14.8 ± 2.80	0.37 ± 0.07	395 ± 114	18 ± 5
BeCyclOp #2	20.2 ± 0.84	0.48 ± 0.02	450 ± 83	16 ± 3

Supplementary Figure 4. Determination of various CyclOps amounts in membranes and BeCyclOp turnover number.

Fluorescence emission of YFP measured for known amounts of purified YFP was used to generate a standard curve (n=3, error bars=SD; upper left panel). Using this standard, amounts of BeCyclOp, CaCyclOp and AmCyclOp1 expressed in oocyte membranes were determined (upper right panel). Furthermore, the BeCyclOp turnover number was determined (lower panel), by measuring BeCyclOp protein amount by fluorescence emission of the YFP tag (n=3). The results are from 2 different batches of oocytes. For #1 and #2, 20 and 21 oocytes were used for membrane extraction and final membrane extracts were suspended to 80 and 84 µL, respectively. 2 µL membrane extracts were used for 1 reaction. Reactions were performed at 20 ℃, error bars = SD.

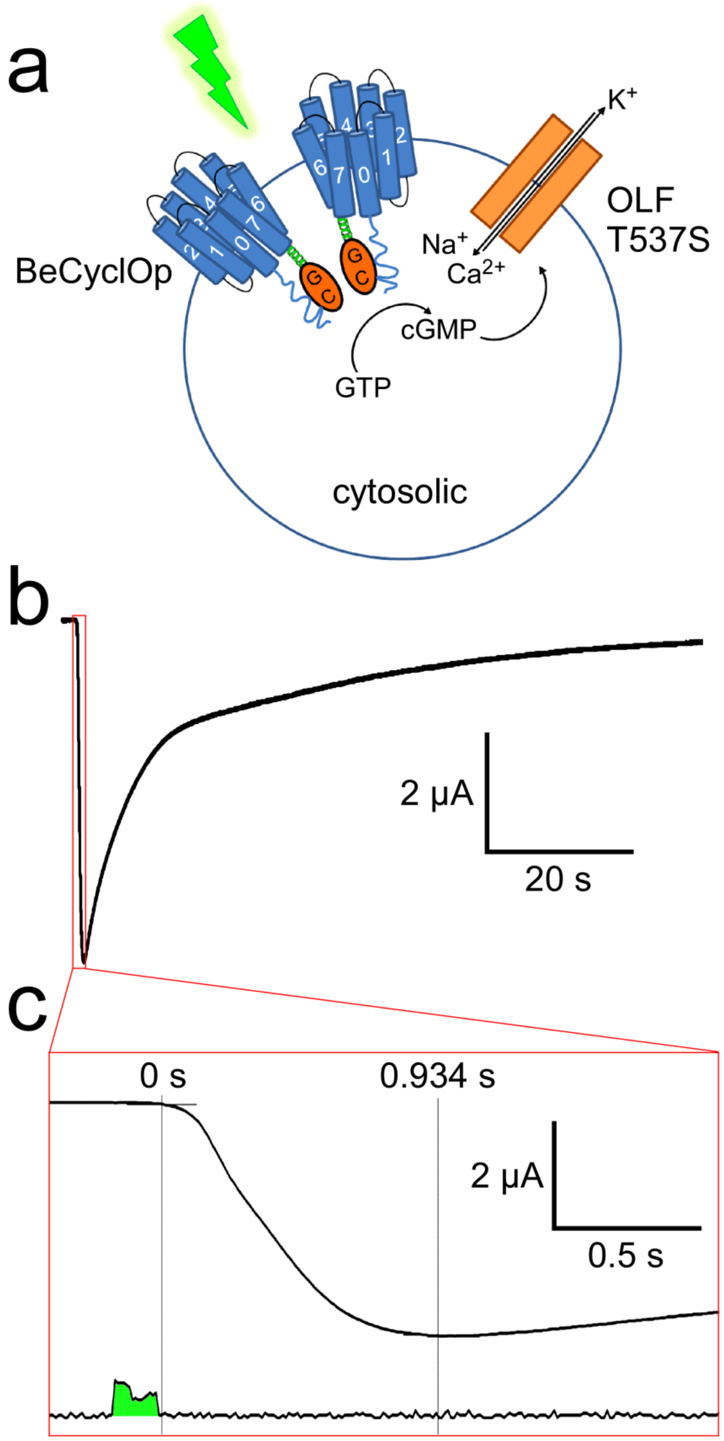

Supplementary Figure 5. Light activation of cGMP-sensitive cation channel OLF/T537S *via* BeCyclOp.

A) Principle for electrophysiological measurement of BeCyclOp induced currents through the co-expressed cyclic nucleotide gated (CNG) channel mutant OLF/T537S. **B**) Photocurrent (at -60 mV) induced by a 100 ms light flash (9 mW mm^{-2}, 532 nm) in *Xenopus* oocyte, 3 days after co-injection of 0.6 ng BeCyclop cRNA and 6 ng OLF/T537S cRNA. **C**) Enlarged current trace, indicating the on- and off-kinetics of the BeCyclOp/OLF system. The upper trace indicates the photocurrent, the lower trace is a record of the voltage driving the 100ms green laser pulse.

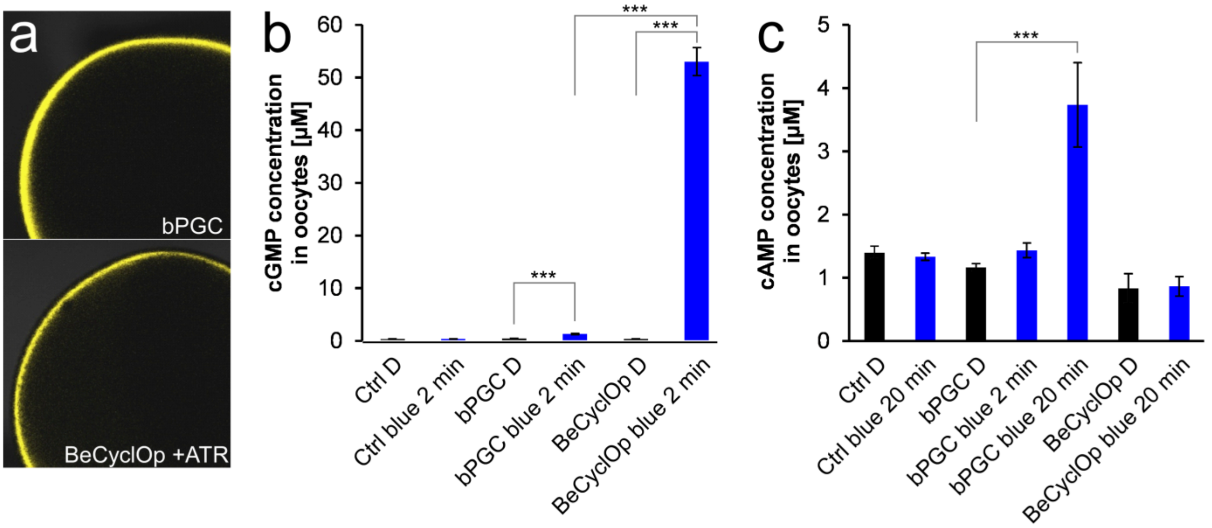

Supplementary Figure 6. BeCyclOp is ~40 fold more efficient in cGMP generation than bPGC (BlgC, EROS).

A) Fluorescence image of *Xenopus* oocytes expressing BeCyclOp::YFP (with 1 µM ATR) and bPGC::YFP. **B**) cGMP production of BeCyclOp and bPGC in *Xenopus* oocytes under dark (D) and 2 min blue light (464 nm, 10 µW mm^{-2}) illumination (L) **C**) Likewise, cAMP production of BeCyclOp and bPGC were determined, as in B). Extract samples are produced 3 days post injection, the injected cRNA amount was ~ 25 fmol for each gene. Shown are the mean values measured from n=3 experiments with 4 oocytes each; error bars = SD. Statistically significant differences determined by 1-way ANOVA: *** P<0.001.

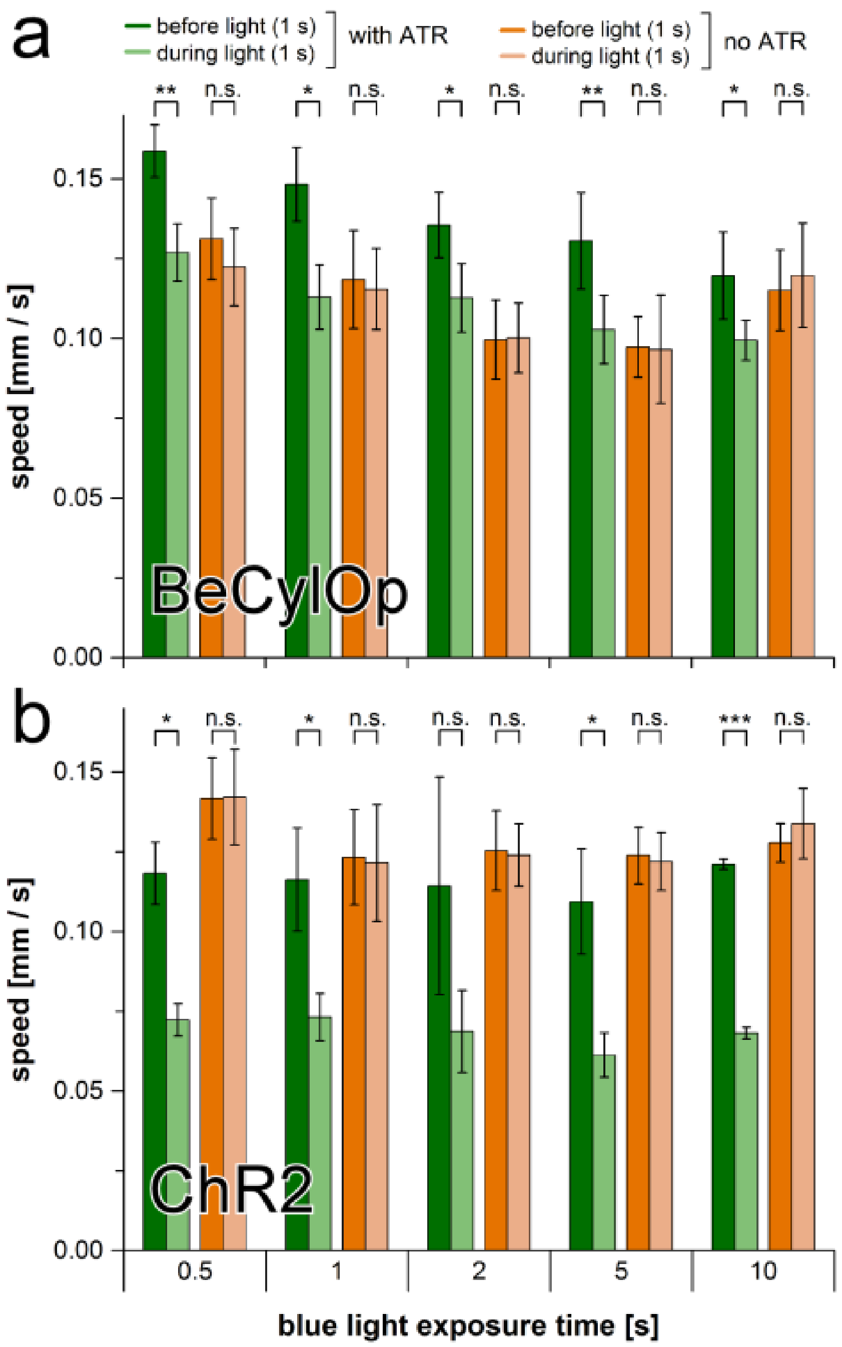

Supplementary Figure 7. Speed changes following the activation of BeCyclOp and ChR2 in CO_2/O_2 sensing BAG neurons of *C. elegans*

A) Average speed 1 second before and after the onset of the blue light illumination of varying durations (0.5, 1, 2, 5, and 10 s) in the locomotion speed assay performed with animals expressing BeCyclOp in BAG neurons in the absence and presence of ATR. N=5(4 for no ATR) experiments with n=15-20 animals each; error bars: SEM. **B**) As in A), but animals expressing ChR2 in BAG neurons were used instead. N=3 experiments with n=15-20 animals each; error bars: SEM. Statistically significant differences determined by paired 2-tailed Student's t-test * P<0.05, ** P<0.01, *** P<0.001.

cGMP concentration	D (μM)	L (μM)
control	0.56 ± 0.03	0.61 ± 0.05
BeCyclOp	0.57 ± 0.01	182 ± 55
BeCyclOp -ATR	0.68 ± 0.17	123 ± 3.5
AmCyclOp1	0.65 ± 0.07	1.05 ± 0.02
AmCyclOp2	0.6 ± 0.07	0.58 ± 0.08
AmCyclOp3	12 ± 4.4	11.8 ± 3.8
CaCyclOp	3.6 ± 0.3	100 ± 25

Supplementary Table 1. In *vivo* assay of different CyclOps in oocytes.

Resulting cGMP concentrations after incubation in the dark. (D), or after 2 min green light (L; 532 nm, 0.15 mW/mm² - BeCyclOp; 511 nm, 0.5 mW/mm² - all other CyclOps).

Supplementary References

1. Krogh, A., Larsson, B., von Heijne, G. & Sonnhammer, E.L. Predicting transmembrane protein topology with a hidden Markov model: application to complete genomes. *J Mol Biol* **305**, 567-580 (2001).
2. Omasits, U., Ahrens, C.H., Muller, S. & Wollscheid, B. Protter: interactive protein feature visualization and integration with experimental proteomic data. *Bioinformatics* **30**, 884-886 (2014).
3. Crooks, G.E., Hon, G., Chandonia, J.M. & Brenner, S.E. WebLogo: a sequence logo generator. *Genome Res* **14**, 1188-1190 (2004).
